# Unexpected High Diversity of Terrestrial Cyanobacteria from the Campus of the University of the Ryukyus, Okinawa, Japan

**DOI:** 10.3390/microorganisms5040069

**Published:** 2017-11-07

**Authors:** Xuan Hoa Nguyen, Shinpei Sumimoto, Shoichiro Suda

**Affiliations:** 1 Graduate School of Engineering and Science, University of the Ryukyus, 1 Senbaru, Nishihara, Okinawa 903-0213, Japan; nxhoa.hua@gmail.com (X.H.N.); e083420@yahoo.co.jp (S.S.); 2 Department of Chemistry, Faculty of Science and Technology, Keio University, 3-14-1 Hiyoshi, Kohoku, Yokohama 223-8522, Japan; 3 Department of Chemistry, Biology and Marine Science, Faculty of Science, University of the Ryukyus, 1 Senbaru, Nishihara, Okinawa 903-0213, Japan

**Keywords:** terrestrial cyanobacteria, diversity, 16S rRNA phylogeny, black stains, campus of the University of the Ryukyus

## Abstract

Terrestrial cyanobacterial strains were isolated from the Nishihara campus of the University of the Ryukyus, Okinawa, Japan. The 13 sampling sites were distributed in a 200 m radius and appeared as dry, blackened stains. From these small areas, 143 cyanobacterial strains were established. The strains were divided into five morphotypes, including unicells, unicells with baeocytes, non-branching filaments, false-branching filaments, and heterocystous strains. From the strains, 105 partial 16S rRNA gene sequences were obtained and could be classified into 30 generic types. Among them, 22 unique strains and over 1100 bps of data were selected for further phylogenetic analyses. These sequences were positioned into six main clades corresponding to cyanobacterial orders: Nostocales, Chroococidiopsidales, Chroococcales, Oscillatoriales, Pleurocapsales, and Synechococcales. Almost all sequences had no identical matching data in GenBank and many of them had no closely related data. These data suggest that the terrestrial cyanobacteria are very divese even within close sampling areas, such as within the campus of the University of the Ryukyus. The established strains are not only important for classification of terrestrial cyanobacteria but also for possible application studies in the future.

## 1. Introduction

Many traditional and even modern buildings are made with white limestone, mortar, and concrete, and such buildings often have black stains, particularly in tropical and subtropical countries. These black stains are sometimes positively considered as atmospheric of past eras for historical monuments, but these stains are usually dealt with as nuisances. These black stains are caused by terrestrial cyanobacterial growth, and many floral studies of such terrestrial cyanobacteria have been reported from many ecosystems in European temperate and Mediterranean regions [1,2,3], India [4,5,6,7], the Middle East [8], Australia [9], China [10], North America [11], Middle and South America including Mexico and Brazil [12,13], the Hawaiian Islands [14], and even from locations with extreme conditions such as Antarctica [15], Arctic Greenland [16], the Alps [17], and Africa [18].

Scientific reports on cyanobacteria in urban areas have appeared in the past few decades. Such black stains have shown to lower values of real estate in Taiwan [19]. Repeated strong radiation and heavy showers give strong light, high temperature, high humidity, and dryness, promoting the growth of black stained terrestrial cyanobacteria [19]. There has been much research on the effects of light and humidity on the growth of epilithic cyanobacteria [20]. From mat populations that form dominating crusts on the surfaces of buildings, roadside walls, rocks, stoneworks, and monuments in South Korea, it was shown that they contained a high diversity of terrestrial cyanobacteria [21,22]. In Japan, terrestrial cyanobacterial studies have been limited; while, globally, 102 species of cyanobacteria have been reported from surfaces of wet soil, rocks, or tree trunks, and 19 species from terrestrial or aerial habitats, in Japan, only *Scytonema ocellatum* has been reported from dry soil ([23], in Japanese). Another report indicates that terrestrial cyanobacteria communities are composed mainly of *Hassallia byssoidea* ([24], in Japanese).

Recently, a number of new genera and taxa have been proposed for cyanobacteria using a polyphasic approach (i.e., [25,26,27]). All of these publications have been based on molecular phylogenetic analyses using at least partial 16S rRNA gene sequences combined with traditional morphological observations. The first estimation for new taxon election or taxonomic evaluation are analyses of phylogenetic positions between types or reference species and the specimen(s) of interest using 16S rRNA gene phylogeny. Subsequently, various characterizations and, in particular, morphological examinations are conducted. Thus, culture strains are very important and useful for the taxonomic treatments of cyanobacteria. However, terrestrial cyanobacterial strains are scarce and limited, even in diversity studies on terrestrial cyanobacteria, as mentioned above. *Hapalosiphon fontinalis* from Hawaii has been reported to contain anti-bacterial and anti-fungal substances [28]. Terrestrial cyanobacteria are considered as one of the promising resource taxa for drug discovery [29]. Many UV-absorbing mycosporine-like amino acids (MAAs) have been reported from the terrestrial cyanobacterium *Nostoc commune* [30]. Such cyanobacterial MAAs may be used in cosmetics [31]. Hence, terrestrial cyanobacteria can also be expected to still contain many discoveries that can be used for a wide variety of applications.

In order to facilitate future utilization, our laboratory has been focusing on basic and applied cyanobacterial research. There have been some studies on terrestrial cyanobacteria growing on urban surfaces of buildings, monuments, and rocks, but no terrestrial cyanobacterial study has been conducted from Okinawa in southern Japan. The same as in Southeast Asian countries, the climate in Okinawa is characterized by high temperatures and humidity, which may be responsible for such cyanobacterial growth on urban buildings and walls, making blackened stains. The specific aim of this study was to clarify the diversity of terrestrial cyanobacteria within a small area, the Nishihara campus at the University of the Ryukyus, and to make culture strains for future studies.

## 2. Materials and Methods

**Figure 1:**
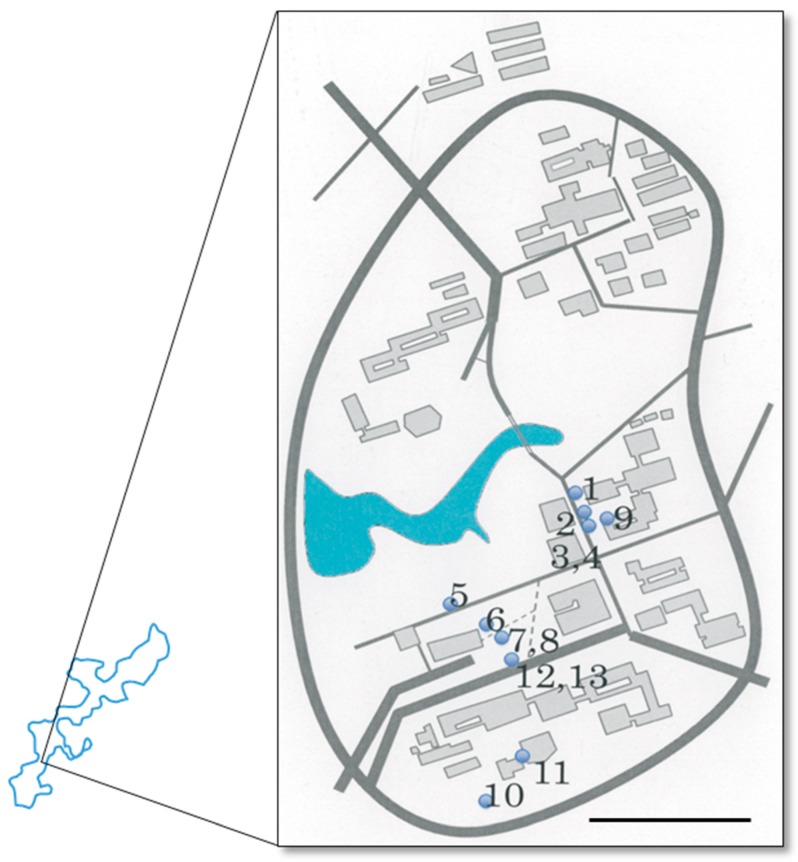
Sampling points at the Nishihara campus of the University of the Ryukyus (circles). 1: Ru1, 2: Ru3, 3: Ru4, 4: Ru5, 5: Ryu1, 6: Ryu2, 7: Ryu3, 8: Ryu4, 9: Ryu5, 10: Ryu7, 11: Ryu8, 12: Ryu9.1, 13: Ryu9.2. Scale bar = 200 m.

All sampling sites were distributed within a 200 m radius inside the Nishihara campus of the University of the Ryukyus (26°14′51.32′′ N, 127°45′55.04′′ E). This is a very small area and can be treated as the same locality. Samples were obtained from the surfaces of buildings, roadside walls, stairs, tree stumps, and monuments within the sampling area (Figure 1). Surfaces of blackened areas were scrubbed with a wet Melamine sponge, or we directly picked up portions of cyanobacterial mats with tweezers.

The sponge or cyanobacterial mats were precultured before isolation in petri dishes using BG11 or BG11-N (without nitrogen) media [32]. Isolations were made by pipette washing method and agar plating. Culture conditions were 22 ± 2 °C, approximately 40 µmol photon m^−2^ s^−1^ provided by cool white fluorescent lamps, with a light and dark cycle of 14L10D. A light microscope (Nikon, Tokyo, Japan) equipped with a SPOT Idea digital camera (Diagnostic Instruments, Inc., Sterling Heights, MI, USA) was used for morphological observations. The morphological classifications followed Komárek and Anagnostidis [33,34] and Komárek [35].

DNA was extracted and purified with a DNeasy Plant mini kit (Qiagen, Japan). Partial 16S rDNA regions were amplified using universal primers 16S27F-16S1494R [36] for 3 strains: Ru3-14(A5), Ru3-1, Ryu2-12 [37], primers CYA106F [38]-16S1541R [39] for strain Ryu8-6, and the cyanobacteria specific primers CYA106F [38]-CYA1371R(1+2+3) [40] for the remaining strains. PCR cycles were set to 94 °C for 5 min, followed by 30 cycles of 94 °C denaturing for 1 min, 60 °C annealing for 1 min, and 72 °C extension for 1 min, with an additional 72 °C extension for 7 min, and an ending hold at 4 °C. The amplified DNA fragments were checked by electrophoresis on a 1% agarose gel with ethidium bromide staining. Amplified PCR products were sent to Macrogen Japan Co. for sequencing.

For phylogenetic analyses, additional data sequences were obtained from NCBI GenBank and used to construct the phylogenetic tree. All sequences were aligned and analysed using MEGA 7.0 [41]. A maximum likelihood phylogenetic tree based on partial 16S rRNA gene sequences was constructed. The General Time Reversible-parameter model (GTR + G + I) was used and the outgroups were selected as *Gloeobacter kilaueensis* (NR_121745) and *Gloeobacter violaceus* PCC 7421 (NR_074282). Bootstrap values were tested 1000 times using the rapid bootstrap option (>50%).

## 3. Results and Discussion

**Figure 2:**
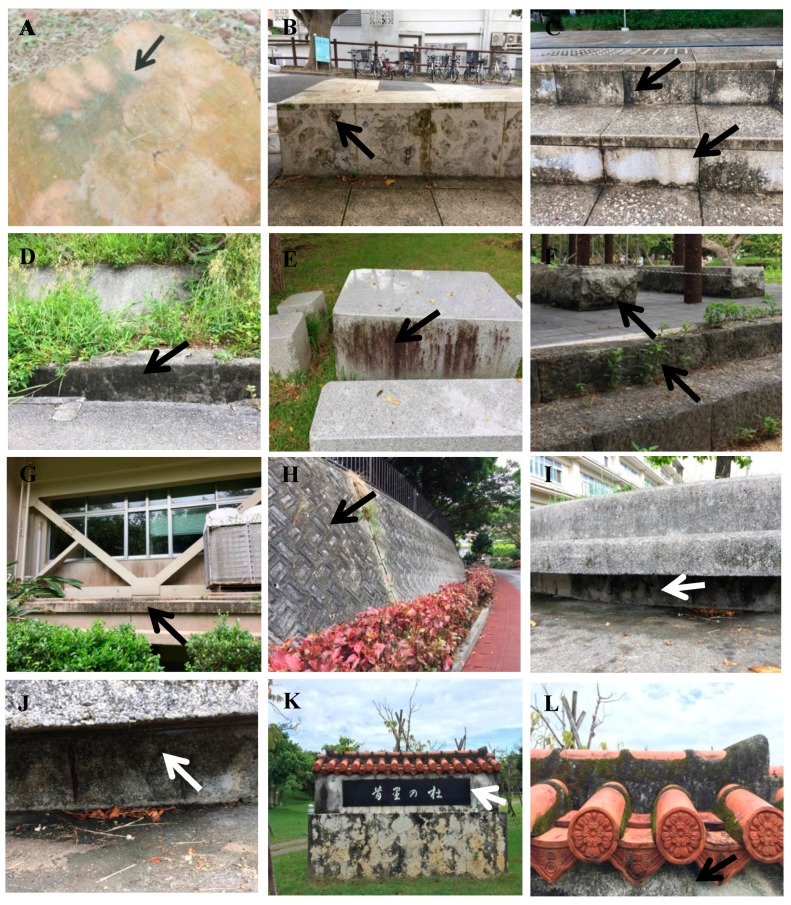
Pictures of blackened sampling points (arrows). (**A**) tree stump, Ru1; (**B**) limestone bench, Ru3; (**C**) limestone stairs, Ru4 and Ru5; (**D**) side wall of ditch, Ryu1; (**E**) side wall of a stone desk, Ryu2; (**F**) side wall of stone foundations, Ryu3 and Ryu4; (**G**) wall of a school building, Ryu5; (**H**) road side wall, Ryu7; (**I**,**J**) bottom part of a concrete bench, Ryu8; (**K**) side of a university monument, Ryu9.1; (**L**) shaded part of the roof of the monument, Ryu9.2.

**Figure 3:**
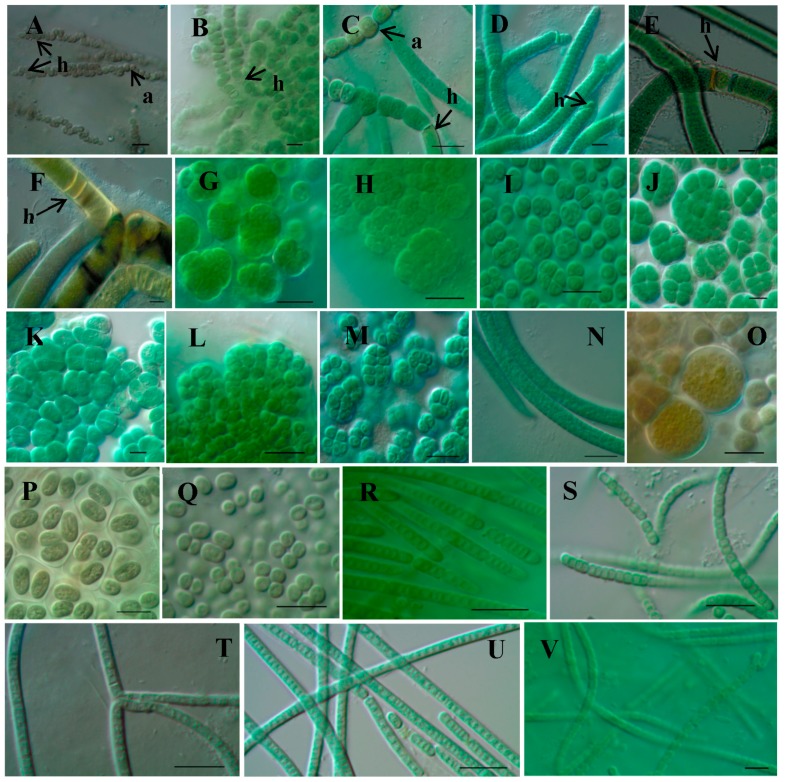
Light micrographs of strains. (**A**) Ru1-3d, (**B**) Ru1-6, (**C**) Ryu2-7DN(D3), (**D**) Ryu5-18(F2), (**E**) Ryu1-11, (**F**) Ryu1A1(C3), (**G**) Ryu4-7, (**H**) Ryu9.1-2, (**I**) Ru3-14, (**J**) Ryu8-6, (**K**) Ryu1-3, (**L**) Ryu2-16, (**M**) Ryu5-15d, (**N**) Ru5-34, (**O**) Ru3-1, (**P**) Ryu1-8DN(B9), (**Q**) Ryu4-4DN(I7), (**R**) Ru3-34, (**S**) Ryu1-2, (**T**) Ru5-44, (**U**) Ryu2-12, (**V**) Ru4-3d. a: akinates, h: heterocysts. Scale bars = 10 µm.

Thirteen samples of mats or scraped Melamine sponges of terrestrial cyanobacteria were collected from within the sampling area (Figure 1), directly obserbed by light microscopy, and pre-cultured for establishing culture strains of terrestrial cyanobacteria. Sampling sites were selected as blackened parts of walls, monument stones, and concrete buildings (Figure 2). Sampling sites were usually dry but the sites had tendencies to be routes for water or became small water puddles or pools during and after rainfall. Sampling sites were also slightly shaded and not under full sunlight. Direct light microscopic observations of the natural samples indicated that the dominant constituents of the samples were *Gloeocapsa* and related chroococcalean types from scraped Melamine sponges, whereas *Scytonema* and related heterocystous filamentous types were dominant in the cyanobacterial mats. After pre-culture, the growing cyanobacteria were isolated by pipette washing method or agar plating to establish culture strains. We established 143 culture strains that belonged to almost all cyanobacterial types, such as unicells, unicells with baeocytes, non-branching filaments, false-branching filaments, and heterocystous strains (Figure 3). From the strains, 16S rRNA gene sequences from morphologically different strains were analysed to obtain 105 sequences belonging to 30 different genetic types. In this study, we eliminated closely related and relatively short (less than 1100 bp) sequences, and finally, 22 strains were selected for diversity evaluation. In this paper, we used 16S rRNA gene data from Komárek et al. [42] and BLAST [43] as a reference for grouping sequences to identify closely related strains (e.g., to at least genus level). Finally, morphological comparisons were done to confirm the identity of strains using Cyanoprokaryota monographs [33,34,35].

### 3.1. Phylogenetic Analyses

**Figure 4:**
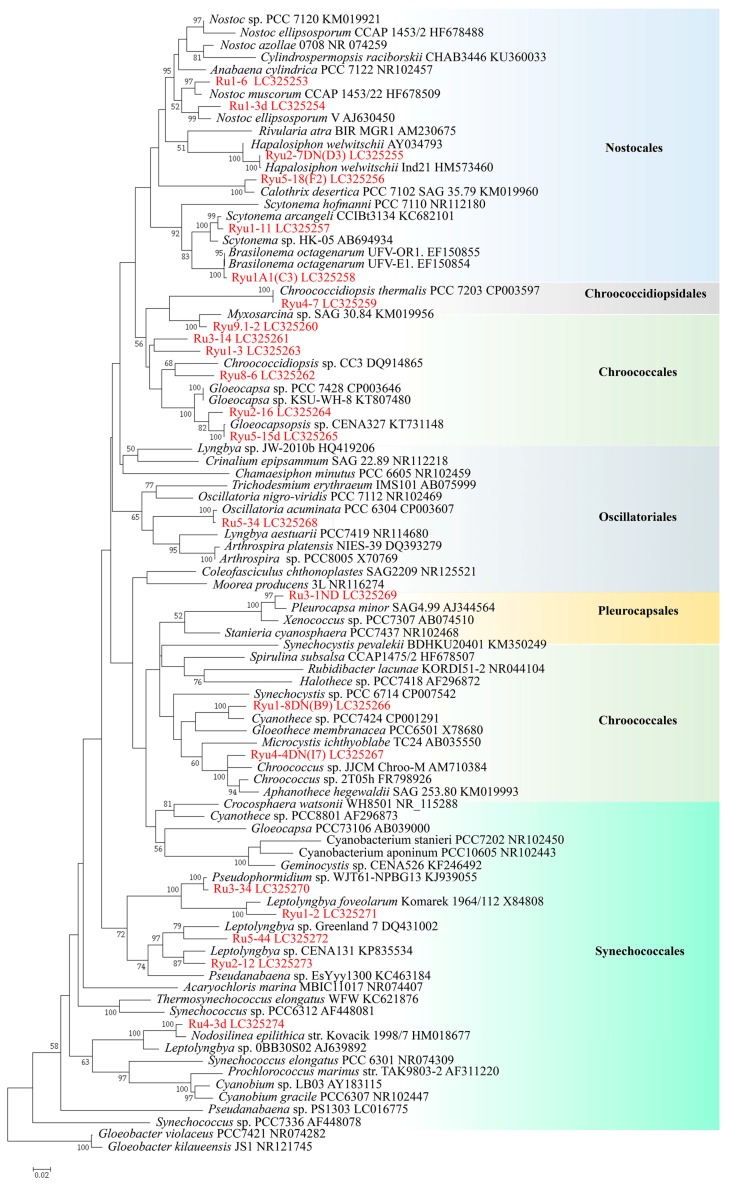
Maximum likelihood tree based on 16S rDNA gene sequences. The General Time Reversible-parameter model (GTR+G+I) was used, with the outgroups selected as *Gloeobacter kilaueensis* (NR121745) and *Gloeobacter violaceus* PCC 7421 (NR_074282). Bootstrap values were tested 1000 times using the rapid bootstrap option (≥50%).

Twenty-two strains (=sequences) approximately 1100 bp in length were selected and utilized in phylogenetic analyses for diversity evaluation of terrestrial cyanobacterial strains. Seventy-one 16S rRNA sequences were obtained from NCBI GenBank from the data of Komárek et al. [42] and from BLAST similarity searches, and were used to construct the phylogenetic tree. We constructed a maximum likelihood (ML) phylogenetic tree with a total of 105 sequences. Bootstrap values were tested 1000 times using the rapid bootstrap option (Figure 4). The resulting phylogenetic tree was divided into six main clades corresponding to Nostocales, Chroococcidiopsidales, Chroococcales, Oscillatoriales, Pleurocapsales, and Synechococcales. Some strains were closely related with GenBank data; e.g., Ryu2-7DN(D3) was closely related with *Hapalosiphon welwitschii* AY034793, Ryu4-7 was closely related with *Chroococcidiopsis thermalis* PCC7203 (CP003597), and Ryu5-15d was closely related with *Gloeocapsopsis* sp. CENA327 (KT731148). Also, many strains were identifiable to the generic levels, some within the same genus; e.g., Ru1-6 and Ru1-3d belonged to the genus *Nostoc*, Ryu1A1(C3) belonged to *Brasilonema*, Ryu1-11 belonged to *Scytonema*, and Ru4-3d belonged to *Nodosilinea*. On the other hand, other strains had no close relatives in GenBank, such as strains Ru3-14, Ryu8-6, and Ryu1-3.

### 3.2. Morphological Comparisons between Molecular Phylogeneticaly Related Strains

All strains require further detailed studies for formal characterization.

#### 3.2.1. Nostocales

**Ru1-3d**: *Nostoc* sp. (Figure 3A, collection point 1)

This strain had heterocysts, with the heterocysts presented at the end of the filament having a rounded, cylindrical, and somewhat conical shape, and the intervening heterocysts being spherical. The diameter of cells was relatively equal, about 4 µm wide, and only one filament was covered with a mucilaginous sheath. We observed that there were two or more splitting faces, with a part of the cells splitting at the face perpendicular to the filamentous body, and the filamentous body had a meandering shape. This *Nostoc* strain was morphologically related with isolates from *Cycas revoluta* and particularly close to *N. punctiforme* or *Nostoc* sp. [35].

**Ru1-6**: *Nostoc* sp. (Figure 3B, collection point 1)

This strain also consisted of heterocysts and was intervening. The diameters of cells were sometimes slightly irregular, 4.1–4.7 µm wide, in young trichomes or, in hormogonia, 6–9.1 µm. Several filamentous bodies were wrapped in a spherical colony covered with a mucilaginous sheath. There were two splitting faces, and some of the cells were in two rows. The cells were olive in color and contained granules inside. Akinetes were not observed. This strain was closely related with *Nostoc edaphicum* Kondrateva, 1962 [35].

**Ryu2-7DN(D3)**: *Hapalosiphon* sp. (Figure 3C, collection point 6)

The strain Ryu2-7DN(D3) (Figure 3C) was genetically closely related with *Hapalosiphonwelwitshchii* AY034793 (Figure 4). This strain had heterocysts and two cell division faces, and the filament were true branches. The branches were almost at right angles to the stem. The stem cells were barrel-shaped, 4.8–5.9 µm width × 5.9–8.5 µm length, and the branch cells were an elongated rod shape. The cells contained granules. Akinetes were continuously produced. In some cases, most of the cells of a filament were akinetes. These akinetes were 5.5–8.0 µm width × 5.3–6.4 µm length. These morphological characters were close to those for *Hapalosiphon welwitschii* W. et G. S. West, 1897, but the cell sizes of the strain were slightly larger than those of *Hapalosiphon welwitschii*. Also, *Hapalosiphon welwitschii* inhabits freshwater streams, different from the strain observed here. Thus, we identified strain Ryu2-7DN(D3) as *Hapaloshiphon* sp.

**Ryu5-18(F2)**: *Tolypothrix* sp. (Figure 3D, collection point 9)

This strain was related with *Calothrix deseritica* PCC 7102 (KM019960) (Figure 4) but the character of having no hairy ends of filaments did not fit with the genus *Calothrix*. In this strain, a heterocyst appeared at the tip of the filament. Filaments had slight narrowing to the tip and no hairy ends, but had false branching, with one filament in one sheath included. Each filament did not form colonies and was isolated. Trichomes were 5–11 µm wide, and dark blue-green or olive-green in coloration. Due to morphological similarity, we identified strain as *Tolypothrix* sp. 

**Ryu1-11**: *Scytonema* sp. 1 (Figure 3E, collection point 5)

Heterocysts were confirmed at both the head and stem parts. Most of the vegetative cells were blue-green, 6.3–9.5 × 10–20 µm wide, but several heterocysts were pale yellow. The filament had a thin sheath. The strain certainly belonged to the genus *Scytonema* by molecular phylogenetic and morphological results.

**Ryu1A1(C3)**: *Brasilonema* sp. 1 (Figure 3F, collection point 5) 

The filament had heterocysts and was mediate. Many filaments were singularly false-branching. Most of the cells were yellowish brown, but several cells were pale white, and 6–9 × 10–20 µm wide. The genus *Brasilonema* has been established by separating from the genus *Scytonema*, based mainly on molecular phylogenetic analyses [43]. Hence, the current strain should be carefully compared with known *Scytonema* species that as of yet have no 16S rRNA gene sequences available publically.

#### 3.2.2. Chroococcidiopsidales

**Ryu4-7**: *Chroococcidiopsis* sp. (Figure 3G, collection point 5)

The planes of cell division had three or more faces to make a three-dimensional cell mass. No baeocytes were confirmed. A large number of cells was covered with a common sheath to form irregular colonies. The strain was identified as *Chroococcidiopsis* sp. [33], and phylogenetically the closest relative was *C. thermalis* PCC 7203, but this species inhabits aquatic environments, different from the current strain that was isolated from a part of dried concrete block.

#### 3.2.3. Chroococcales

**Ryu9.1-2**: *Cyanosarcina* sp. (Figure 3H, collection point 12)

This strain showed regular division in young cells, and colonies had a cuboidal shape and were blue-green. In addition, the cell size was 3.8 ± 0.4 µm (*n* = 30), which indicates this strain could be identified as *Cyanosarcina* sp. [33]. The phyloenegetic positon of the strain was sister to *Myxosarcina* sp. SAG 30.84 in the order Chroococcales, and there was no closely related sequence (Figure 4).

**Ru3-14**: (Figure 3I, collection point 2)

Cells divided regularly. Grown colonies had a spherical shape, and cells were dispersed. The diameter of the cells were 2.3–10 µm. These characters agreed with *Pseudocapsa* [33] but there was no closely related sequence in GenBank.

**Ryu8-6**: *Pseudocapsa* sp. (Figure 3J, collection point 11)

Cells divided regularly and young colonies took on a feathered or radial arrangement, indicating a close relationship with *Pseudocapsa dubia* [33]. In old colonies, extracellular polysaccharides were also found to be colored yellowish brown. The diameter of the cells was 7.5 ± 0.5 μm (*n* = 30). The strain was not closely related to any sequence in GenBank. 

**Ryu1-3**: *Cyanosarcina* sp. (Figure 3K, collection point 5)

Cells were spherical or elliptical, with 1–4 cells present in the original sheath, 13–23 μm in diameter. Cell divison was continuous in multiple faces without growing to the shape and size of the original cell, and hemispherical and quarter-sphere cells were also common. Many of these cells were wrapped in thin sheaths to form colonies. The strain was closely related with *Cyanosarcina* sp. but there was no related sequence in GenBank. 

**Ryu2-16**: *Gloeocapsopsis* sp. (Figure 3L, collection point 6)

Cells had various forms such as spherical, elliptical, and polygonal shapes, etc. Each individual cell was in a sheath or unwrapped. A number of cells formed amorphous massive colonies and some cells were packed in a mucilaginous sheath. There were numerous cell division faces, and after division, the cells did not grow to their original shape and size but still were carrying out the next division. The strain had no close sequence in GenBank.

**Ryu5-15d**: *Gloeocapsopsis* sp. (Figure 3M, collection point 5)

Cells divided continuously and regularly, similar to the form of *Gloeocapsopsis*. The grown colonies showed a spherical or oval shape, and cells were dispersed by the sheath, becoming pale. The diameter of the cells was 3.8–8.8 µm width × 4.6–12.5 µm length. The strain’s sequence was closely related with *Gloeocapsopsis* sp. CENA327 (KT731148).

**Ryu1-8DN(B9)**: *Gloeothece* sp. (Figure 3P, collection point 5)

The cells were rod-shaped. One cell or a plurality of cells were covered with a definite sheath to form colonies. Although the spacings between cells were narrow, cells did not adhere tightly. Granules were observed in the cells. Cell color was dirty greyish-green or pale blue-green, and cell dimensions were 4.8–5.2 µm wide × 6–9.2 µm long. The strain was closely related with *Gloeothece tepidariorum* [33].

**Ryu4-4ND(I7)**: *Aphanocapsa* sp. (Figure 4Q, collection point 8)

Cells were elliptical or spherical and slightly elongated, 2.8–3.3 µm wide × 3–5.1 µm long. A large number of cells were wrapped in a mucilaginous sheath to form colonies, but each cell did not clump. One to four cells generated by division sometimes gathered together, but they did not touch each other, and there were some distances between other cells. The cell divisions were probably on three faces. After division, the cells grew to their original size and shape and then proceeded to the next division. Morphologically the strains were closely related with *Aphanocapsa parietina* Nageli 1849 [33], but there is no authentic data for this genus in GenBank.

#### 3.2.4. Oscillatoriales

**Ru5-34**: *Phormidium* sp. (Figure 3N, collection point 4)

This strain was closely related with *Oscillatoria acuminata* PCC 6304 (CP003607) (Figure 4). The filamentous body did not have a heterocyst or calyptora. Each filament did not have a sheath. Each cell had a rectangular shape with a short side parallel to the filamentous body, and the tip cells at the end were narrowing and bent, 1.9–3.2 μm long × 5.5–7.5 μm wide. In addition, cells had gliding motility with active rotation. These characters agree well with many morphologically related species in *Phormidium* [34], and further comparative studies are required.

#### 3.2.5. Pleurocapsales

**Ru3-1ND**: *Chroococcopsis* sp. (Figure 3O, collection point 2)

Cells were spherical, elliptical, polygonal, etc., and varied in size. Additionally, the cells showed polarity. There were numerous cell division faces; growth by endospores (baeocytes), and their parental sheaths did not remain after divisions. The cells were yellowish brown, and 15–18 μm in diameter. These morphological characters agree with *Chroococcopsis* [33], but there is no authentic data for this genus in GenBank.

#### 3.2.6. Synechococcales

**Ru3-34**: *Pseudophormidium* sp. (Figure 3R, collection point 2)

Filaments were solitary or in small groups, composed of thin, colourless sheaths, which were sometimes slightly telescopically widened at the ends. False branchings were not very common. Trichomes were not constricted at cross-walls and were slightly attenuated towards the ends, and rare, screw-like coiled sheaths were observed. Cells were blue-green, shorter in length than width, 1.2–1.6 μm long × 2.3–2.6 μm wide. *Pseudophormidium batrachospermi* (Starmach) Anagnostidis et Komarek 1988 was morphologically related [34] but there are no data for this species in GenBank.

**Ryu1-2**: *Leptolyngbya* sp. (Figure 3S, collection point 5)

In this strain, short filaments were densely entangled to form a thin mat that adhered to the substrate. In addition, grown colonies had a bundle shape with filiforms tangled. The sheath was thin, colorless, and adhered to the filamentous body. The filament was slightly curved, resulting in many filaments being tangled. False branching was not abundant. Cells were blue-green and clearly constricted at the cross walls. Cells at the terminal were rounded, the width of the cells was 1.9 ± 0.1 μm, and the length was half to the same as the width, 1–2.5 μm. From these morphological features, the strain was thought to be closely related with *Leptolyngbya henningsii* (Lemmermann) Angnostidis 2001 [34].

**Ru5-44**: *Leptolyngbya* sp. (Figure 3T, collection point 4)

The Ru5-44 strain had a bundle shape with long, tangled colonies that did not adhere to the substrate. The filament was straight, slightly wavy or coil-like, and frequently branched. The sheath was colorless, thin, and adhered to the cell. The cells were light bluish green, and the cells were not constricted. Terminal cells were rounded at the ends but never pointed apart. The width of cells was 2.2–2.8 μm, which was slightly shorter than the normal width [34]. 

**Ryu2-12**: *Leptolyngbya* sp. (Figure 3U, collection point 6)

Filaments were curved, densely entangled, sometimes pseudobranched, and 1.1–1.9 μm long × 2.5–3.2 μm wide; pseudobranches geminate, thinner than main filaments, nearly whip-like. Sheaths were thin and colourless. Trichomes were pale blue-green to almost clear, (1) 1.3–2 (3) μm wide, and strongly constricted at the ungranulated cross-walls. Cells were isodiametric or somewhat shorter or longer than wide in the main filaments, and somewhat longer than wide in the pseudobranches. Apical cells were rounded. Morphologically the strain was related with *Leptolyngbya boryana* Anagnostidis et Komarek 1988 [34].

**Ru4-3d**: *Nodosilinea* sp. (Figure 3V, collection point 3)

The filamentous body did not have ant heterocysts, and no calyptra was confirmed. The sequence was sister to that of *Nodosilinea epilitheca* Perkerson & Casamatta [44] (Figure 4), and morphologically the strain agreed well with this genus [42], but further studies are required to confirm this.

In conclusion, these results and data suggest that terrestrial cyanobacteria is very diverse in Okinawa, even within a small area such as inside the University of the Ryukyus campus. In this study, we suceeded in establishing 143 strains from 13 samples. A total of 105 partial 16S rRNA gene sequences were obtained, and they were divided into 30 generic types of six main cyanobacterial order clades [40]. This means that the terrestrial cyanobacterial diversity at the University of the Ryukyus campus is more diverse than in locations from other surveys, including in India [4,5,6,7], South Korea [21,22], the main Haiwaiian islands [14], Brazil, and Mexico [12,13].

Many archaeologically important stone temples and mortar monuments with artistic structure, as well as the surfaces of painted buildings, are disfigured by changed colour surfaces and caused aesthetic and structural damage due to the colonization of cyanobacterial biofilms [7,43,45]. The diversity distribution of particular cyanobacteria on monuments in urban regions are thought to be related mostly to environmental conditions of the climate, such as water availability and air circulation, and to the position and orientation of the hard surface [43]. Correlations among other environmental factors have been tested to identify other factors that could substitute for the effects of humidity and light intensity [22]. To examine such environmental factors, it is likely that selecting a few sampling points in the Nishihara campus of the University of the Ryukyus should be enough due to the very high diversity of terrestorial cyanobacteria at this location. 

In this study, 22 selected strains were used for phylogenetic analyses. From the results, no identical sequence data were present in GenBank, and surprisingly, many strains’ sequences had no closely related data. Among these strains, some could be identified to generic level by morphology, and hence it is required to study in detail both the molecular phylogeny and morphology of each strain. Finally, we successfully established a very diverse, 143 terrestrial cyanobacterial strain collection, and the collection may contain useful strains for applications in the future.

## 4. Conclusions

We collected terrestrial cyanobacteria from blackened parts of walls, monument stones, and concrete buildings at a total of 13 sampling sites in the Nishihara campus at the University of the Ryukyus (Figure 1 and Figure 2). The dominant constituents of the samples were *Gloeocapsa* and related chroococcalean types from scraped Melamine sponges, whereas *Scytonema* and related heterocystous filamentous types were dominant in the cyanobacterial mats. We used BG11 and BG11-N agar plating to attempt to establish culture strains from all 143 isolated strains belonging to almost all cyanobacterial types (Figure 3). From morphologically different strains, 16S rRNA gene sequences were analysed to obtain 105 sequences belonging to 30 different genetic types. Twenty-two strains (=sequences) of approximately 1100 bp in length were selected and utilized in phylogenetic analyses for a diversity evaluation of terrestrial cyanobacterial strains with 71 16S rRNA sequences obtained from NCBI GenBank/BLAST and the data of Komárek et al. [42]. The resulting phylogenetic tree was divided into six main clades corresponding to Nostocales, Chroococcidiopsidales, Chroococcales, Oscillatoriales, Pleurocapsales, and Synechococcales. Also, many strains were identifiable to the generic level, with some within the same genus; e.g., Ru1-6 and Ru1-3d, Ryu1A1(C3), Ryu1-11, and Ru4-3d. On the other hand, other strains had no close relatives in GenBank, such as strains Ru3-14, Ryu8-6, and Ryu1-3. These results provide useful baseline diversity data for research in the future.
